# Molecular and cellular characterization of immunity conferred by lactobacilli against necrotic enteritis in chickens

**DOI:** 10.3389/fimmu.2023.1301980

**Published:** 2023-11-07

**Authors:** Mohammadali Alizadeh, Bahram Shojadoost, Nitish Boodhoo, Sugandha Raj, Shayan Sharif

**Affiliations:** ^1^ Department of Pathobiology, Ontario Veterinary College, University of Guelph, Guelph, ON, Canada; ^2^ Ceva Animal Health Inc., Guelph, ON, Canada

**Keywords:** necrotic enteritis, lactobacilli, chicken, cytokine, lymphocyte, microbiome

## Abstract

Necrotic enteritis is an important enteric disease of poultry that can be controlled with in-feed antibiotics. However, with the concerns over antimicrobial resistance, there is an increased interest in the use of alternatives. Probiotics are one of the alternatives that have gained considerable attention due to their antimicrobial and immunomodulatory activities. Therefore, in the present study, we evaluated the effects of two different *Lactobacillus* species alone or as a cocktail on prevention of necrotic enteritis. Day-old male broiler chickens were divided into five groups and on days 1, 8, 15, and 22, birds in groups 2 and 3 received 1×10^8^ colony forming units (CFU) of *L. johnsonii* and *L. reuteri*, respectively. Group 4 received probiotic cocktails containing both bacteria (10^8^ CFU/bird) and the negative and positive control groups did not receive any lactobacilli. Starting on day 23 post-hatch, birds in all groups (except the negative control group) were orally challenged twice per day with 3×10^8^ CFU of a pathogenic *C. perfringens* strain for 3 days. Tissue and cecal samples were collected before and after challenge to assess gene expression, lymphocyte subsets determination, and microbiome analysis. On day 26 of age, lesion scoring was performed. The results demonstrated that the group that received the lactobacilli cocktail had significantly reduced lesion scores compared to the positive control group. In addition, the expression of interleukin (IL)-12 in the jejunum and CXC motif chemokine ligand 8 (CXCL8), IL-13, and IL-17 in the ileum were downregulated in the group that received the lactobacilli cocktail when compared to the positive control. Treating chickens with the lactobacilli cocktail prior to challenge enhanced the percentage of CD3^-^CD8^+^ cells and Bu-1^+^IgY^+^ B cells in the ileum and increased the frequency of monocyte/macrophages, CD3^-^CD8^+^ cells, Bu-1^+^IgM^+^, and Bu-1^+^IgY^+^ B cells in the jejunum. Treatment with the lactobacilli cocktail reduced the relative expression of Gamma-Protobacteria and Firmicutes compared to the positive control group. In conclusion, the results presented here suggest that treatment with the lactobacilli cocktail containing *L. johnsonii* and *L. reuteri* reduced necrotic enteritis lesions in the small intestine of chickens, possibly through the modulation of immune responses.

## Introduction

Necrotic enteritis (NE) is a multifactorial enteric disease of poultry caused by the overgrowth of pathogenic *Clostridium perfringens* (CP) and is characterized by inflammation, multifocal hemorrhages, and necrosis in the mucosa layer of the small intestine of chickens (1). CP is a Gram-positive, anaerobic, spore-forming bacterium that is a natural inhabitant of the gastrointestinal tract in most avian species (2). Some of these bacteria may contain toxicogenic genes. The overgrowth of these CP strains under certain conditions leads to the production of several tissue-degrading and pore-forming toxins and causes NE (3). As an opportunistic pathogen, the overgrowth of CP occurs in the presence of several nutrition- and environment-related predisposing factors, including a diet containing high levels of non-starch polysaccharides and crude protein, high humidity, high stocking density, reduced ventilation, and poor litter condition, in addition to other stress factors that can suppress the immune system of chickens and disturb the balance of intestinal microbiota (4, 5). The overgrowth of CP leads to the expression of various virulence factors by this bacterium, including bacteriocins, enzymes, adhesion molecules and tissue-degrading toxins, such as NE B-like toxin (NetB), α-toxin, and TpeL (6, 7). Although the clinical form of NE is associated with high morbidity and mortality in broiler flocks, subclinical NE results in decreased production output in poultry and significant economic losses by damaging the intestinal mucosa, leading to impaired nutrient digestion and absorption and reduced overall performance (8–10).

In past decades, NE has been prevented and controlled using in-feed antibiotics. However, with public health concerns over the emergence of antimicrobial resistance in bacteria, the use of antibiotics has been banned or limited in many countries (4, 11). Therefore, several alternatives to antimicrobials have been introduced in the poultry industry to help maintain the health and productivity of broiler flocks given the challenges associated with CP-induced NE in the post-antibiotic era (12, 13).

Probiotics are one of the alternatives that have gained considerable attention because of their potential antimicrobial and immunomodulatory properties (14). Probiotics exhibit beneficial effects in subclinical NE via different mechanisms in chickens (15). The mechanisms of action of probiotics include competitive exclusion, modulation of mucosal immune responses that enhance resistance to CP-associated virulence factors, production of antimicrobial peptides, release of short-chain fatty acids that lower intestinal pH and inhibit CP growth, and restoration of the gut microbiota composition, preventing gut dysbiosis (13, 16, 17).

There is some evidence that probiotics can modulate innate mucosal immune responses in chickens and prevent subclinical NE in experimental challenge models (15, 18). In addition, *in vitro* findings have also demonstrated that lactobacilli reduces CP growth and α-toxin production (19). Therefore, the present study was conducted to evaluate the effects of individual and combined administration of *L. johnsonii* and *L. reuteri* on the gut mucosal immune system and CP-induced NE in broiler chickens.

## Materials and methods

### Animal housing, experimental design and sampling

One-day-old male broiler chickens (n=90) obtained from a commercial hatchery (Guelph, Canada) were randomly assigned to five treatment groups. Birds were group housed in separate floor pens with wood shaving in the Isolation Facility of the Ontario Veterinary College, University of Guelph. Birds in groups 2, 3, and 4 were orally inoculated with 10^8^ colony forming unit (CFU)/bird of different *lactobacillus* species at day 1 before placing them on their designated pens as well as day 8, 15, and 22 post-hatch. Groups 2 and 3 received *L. johnsonii* and *L. reuteri*, respectively, and group 4 received a probiotic cocktail containing both species. Group 1 and 5 served as negative and positive control groups and did not receive lactobacilli (**Table 1**). On days 23 to 25 post-hatch all birds in groups 2, 3, 4, and 5 were orally challenged with 3 x 10^8^ CFU/ml of a highly pathogenic strain of CP (CP4) twice daily. Group 1 remained unchallenged. On day 26, all birds were euthanized, and lesion scoring was performed. On days 23 (before CP challenge) and 26 post-hatch, tissue samples from jejunum and ileum (6 birds per group) were collected in RNAlater (Thermo fisher scientific Mississauga, ON, Canada) and stored at -80°C for the cytokine gene expression. Segments of jejunum and ileum (5 cm) were collected and stored on ice in PBS containing penicillin-streptomycin for flow cytometry analysis. Cecal contents were collected for analysis of bacterial composition at the phylum level. All animal experiments were approved by the University of Guelph Animal Care Committee according to the guidelines for the use of animals (AUP#4051).

**Table 1:** Experimental Groups.

Group	Lactobacilli oral inoculation at days 1, 8, 15, and 22	C. perfringens challenge at days 23-25
1- Negative Control (NC)	–	–
2- L. johnsonii (L.j)	+	+
3- L. reuteri (L.r)	+	+
4- L.j + L.r	+	+
5- Positive Control (PC)	–	+

### Lactobacilli species isolates, culture conditions and screening

*Lactobacillus spp* including *L. reuteri-*JB/SL-42 and *L. johnsonii-*JB/SL-39 were previously isolated from the intestines of healthy broiler chickens and have been characterized by 16S RNA sequencing. The lactobacillus species used in this study were selected because of their inhibitory activities against CP using *in vitro* screening tests such as agar well diffusion assay and co-culture assay (unpublished data). *Lactobacillus* species were grown anaerobically at 37°C overnight in de Man, Rogosa and Sharpe broth medium (Sigma Aldrich, Oakville, ON, Canada). Following centrifugation at 3000 X g for 10 min, bacterial cells were collected, washed (2X) and resuspended in PBS. Optical densities (OD) were measured at 600 nm by a spectrophotometer (Thermo Fisher Scientific, Mississauga, ON, Canada) and the desired bacterial cell densities (10^8^ CFU/ml) were prepared. An equal amount of each individual species was mixed to obtain 10^8^ CFU/ml of the lactobacilli cocktail.

### Experimental induction of necrotic enteritis

NE was reproduced as previously described (20). On day 14 post-hatch the starter diet was changed, and chickens were provided with a wheat-based diet with high levels of crude protein (30%) and non-starch polysaccharides. A highly pathogenic strain of CP (CP4) was used in this study that had been generously provided by Dr. John Prescott (University of Guelph). For challenging chickens, CP bacteria were primarily grown in Cooked Meat Medium (ThermoFisher Scientific, Mississauga, ON, Canada) anaerobically at 37°C overnight. The growth medium was then added to fluid thioglycolate medium (FTG; Sigma Aldrich, Oakville, ON, Canada) and incubated for 15 h at 37°C in aerobic condition. On day 23 post-hatch, birds were orally challenged with an inoculum containing 3 x 10^8^ CFU/ml of CP in the morning (9 am) and afternoon (3 pm) for three consecutive days (23-25). On day 26 post-hatch, all chickens were euthanized, and lesions were scored on a scale of 0 to 6 as previously described (21).

### RNA extraction, reverse transcription, and bacterial DNA extraction

Total RNA was extracted form jejunum and ileum using Trizol (ThermoFisher Scientific, Mississauga, ON, Canada) as described previously (22). RNA was treated with DNase and quantity and quality of the RNA was assessed using a Nanodrop spectrophotometer (ThermoFisher Scientific, Mississauga, ON, Canada). Reverse-transcription to complementary DNA (cDNA) was carried out using Superscript® II First Strand Synthesis kit (Invitrogen, Burlington, ON, Canada) according to the manufacturer’s protocol. For cecal contents samples, microbial genomic DNA was extracted using QIAamp® Fast DNA Stool Mini Kit (Qiagen, Mississauga, ON, Canada) and DNA concentration was measured and adjusted to 40 ng/µL using a Nanodrop spectrophotometer. Sequences of the primers used for PCR reactions and annealing temperatures are shown in **Table 2**.

**Table 2:** Primer sequences used for real-time quantitative PCR (2).

Gene^2^	Primer sequence^3^ (5’-3’)	Annealing temperature	Reference
*IFN-γ*	F: ACACTGACAAGTCAAAGCCGCACA R: AGTCGTTCATCGGGAGCTTGGC	60	St Paul et al. (2011)
*CXCL8*	F: CCAAGCACACCTCTCTTCCA R: GCAAGGTAGGACGCTGGTAA	64	St Paul et al. (2011)
*IL-10*	F: AGCAGATCAAGGAGACGTTC R: ATCAGCAGGTACTCCTCGAT	60	St Paul et al. (2011)
*IL-12p40*	F: CCAAGACCTGGAGCACACCGAAG R: CGATCCCTGGCCTGCACAGAGA	60	St Paul et al. (2012)
*IL-13*	F: ACTTGTCCAAGCTGAAGCTGTC R: TCTTGCAGTCGGTCATGTTGTC	60	St Paul et al. (2011)
*IL-17*	F: TATCAGCAAACGCTCACTGG R: AGTTCACGCACCTGGAATG	64	Crhanova et al. (2011)
*β-Actin*	F: CAACACAGTGCTGTCTGGTGGTA R: ATCGTACTCCTGCTTGCTGATCC	58	St Paul et al. (2011)
*Universal*	F: AAACTCAAAKGAATTGACGG R: CTCACRRCACGAGCTGAC	61	Bacchetti De Gregoris (2011)
*Firmicutes*	F: TGAAACTYAAAGGAATTGACG R: ACCATGCACCACCTGTC	61	Bacchetti De Gregoris (2011)
*Bacteroidetes*	F: CRAACAGGATTAGATACCCT R: GGTAAGGTTCCTCGCGTAT	61	Bacchetti De Gregoris (2011)
*γ-Proteobacteria*	F: TCGTCAGCTCGTGTYGTGA R: CGTAAGGGCCATGATG	61	Bacchetti De Gregoris (2011)
*Actinobacteria*	F: TACGGCCGCAAGGCTA R: TCRTCCCCACCTTCCTCCG	61	Bacchetti De Gregoris (2011)

^1^The listed oligonucleotides were used to analyze the gene expression via real-time quantitative PCR.

^2^IFN, Interferon; IL, Interleukin.

^3^F, forward; R, reverse.

### Real-time reverse transcriptase polymerase chain reaction

The LightCycler 480 II system (Roche Diagnostics GmbH, Mannheim, DE) was used to perform quantitative real-time PCR, as previously described (23). The relative expression of interferon (IFN)-γ, interleukin (IL)-8, IL-10, IL-12p40, IL-13, and IL-17, to the housekeeping gene β-actin was calculated. The cecal bacterial abundance at phylum-level including Actinobacteria, Firmicutes, γ-Proteobacteria, and Bacteroidetes to universal primers was assessed using qRT-PCR as well.

### Intestinal mucosal mononuclear cells preparation and flow cytometry analysis

Tissues from the jejunum and ileum were collected from all treatment groups (n=6 birds/group) and stored on ice in PBS containing penicillin-streptomycin (1%). Tissues were cut into small segments (0.5 cm^2^) and washed thrice with PBS. To facilitate the isolation of mononuclear cells from intestine segments, tissues were incubated (37°C) in PBS containing collagenase type 1 (Millipore-Sigma, Canada) at concentration of 80 units/ml for 20 min. Tissues were then filtered through 40-μm cell strainers (Fisher Scientific, Mississauga, ON, Canada) using the rubber end of a 10-ml syringe plunger. Jejunum and ileum cell suspensions were overlaid (2:1) onto Histopaque-1077 (Sigma, Oakville, ON) and centrifuged at 2100 rpm (600 x g) for 20 min at 21°C to allow density gradient separation. Mononuclear cells were then harvested at the interface and washed twice with RPMI medium (Invitrogen, Burlington, Ontario, Canada) containing 10% fetal bovine serum (Millipore-Sigma, Canada) and 1% penicillin-streptomycin. Cells were counted using a hemocytometer and trypan blue exclusion method and resuspended in 96-well plates at a density of 5 x 10^6^/ml in RPMI. Cells were washed (2X) in fluorescent activated cell sorting (FACS) buffer (PBS containing 1% bovine serum albumin) and stained for 30 min at 4°C in the dark with fluorescent monoclonal antibodies. Two different surface staining panels were used for this study. Panel 1: mouse anti-chicken CD3-PB, mouse anti-chicken CD4-PE-Cy7, mouse anti-chicken CD8-APC, mouse anti-chicken monocyte/macrophage-FITC (KUL01), and mouse anti-chicken TCRγδ-PE (TCR-1). Panel 2: mouse ani-chicken Bu-1-PB, mouse anti-chicken IgM-APC, mouse ani-chicken IgY-FITC antibodies were obtained from SouthernBiotech (SouthernBiotech, Birmingham, AL, USA). For exclusion of the dead cells in both staining panels, the fixable Live/Dead near-Infrared fluorescent reactive dye (ThermoFisher Scientific, Canada) was used. Subsequently, cells were washed in FACS buffer (2X) and fixed in 2% paraformaldehyde. Fixed cells were acquired using FACS Canto II flow-cytometer (BD Bioscience, San Jose, CA, USA) and FlowJo software (v.10) was used for data analysis. The gating strategies are shown in **Supplementary Figure 1**.

### Data analysis

Data were analyzed using one-way ANOVA (GraphPad Prism-version 9) followed by Tukey’s multiple comparison test to determine differences among means. Shapiro–Wilk test was used to verify normal distribution of data. For data that were not-normally distributed, a non-parametric test (Kruskal-Wallis) was used followed by Dunn’s test. Log transformation was performed when error deviation did not have homogeneous various across the treatment groups. Results were considered statistically significant when *P*-value was less than 0.05.

## Results

### Gross necrotic lesions

**Figure 1:**
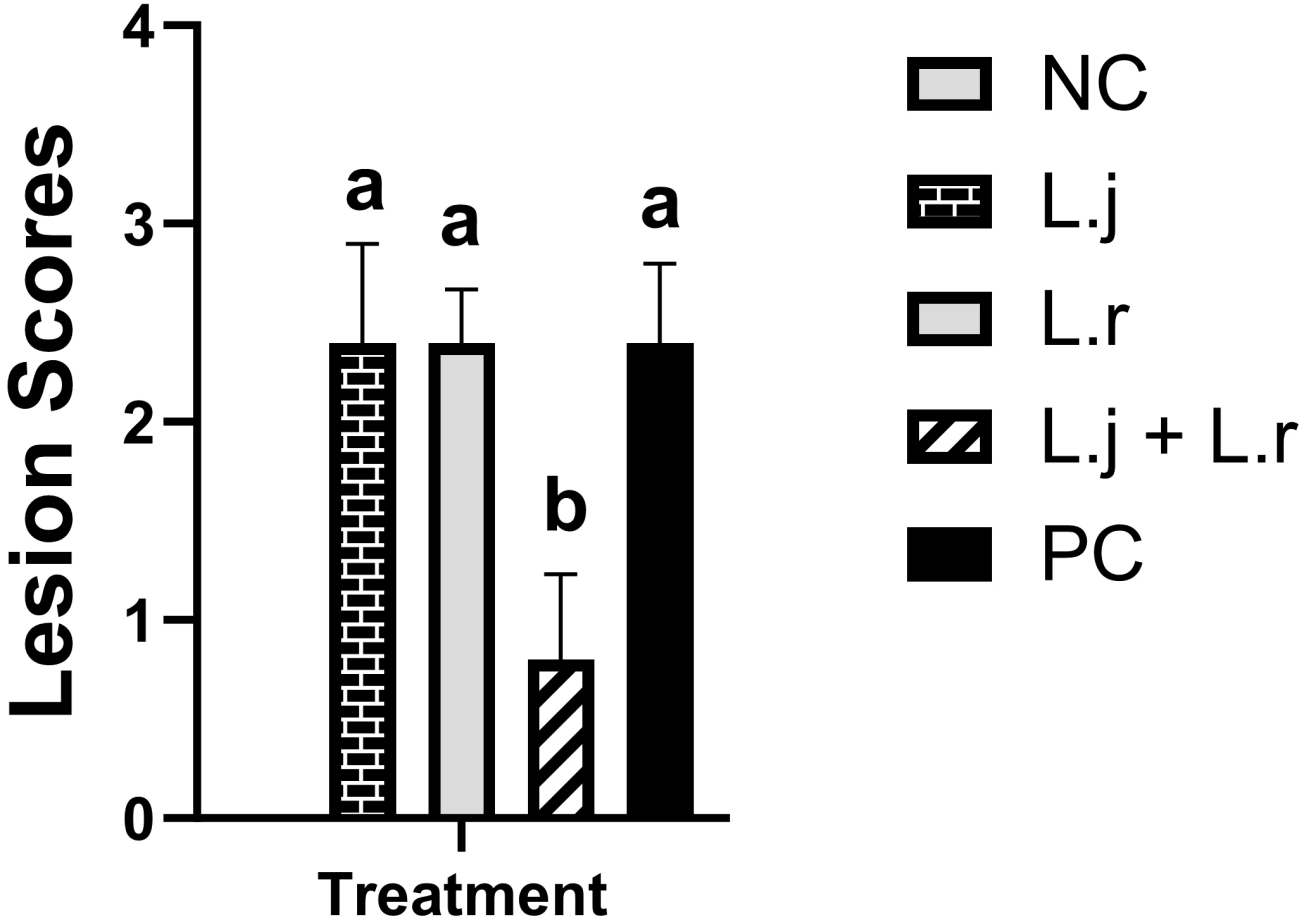
Intestinal Lesion Scores Data represent intestinal lesion scores in birds. On day 26 post-hatch, 10 birds per group were euthanized, and lesion scoring was performed. Group means with no common alphabetical letters differ significantly. Error bars represent the standard error of the mean. Results were considered statistically significant if *P* < 0.05.

The results for gross pathology (**Figure 1**) showed that although no significant difference was observed for groups that received *L. johnsonii* or *L. reuteri* alone, oral inoculation of the lactobacilli cocktail containing both species significantly reduced (*P* < 0.05) mean gross lesion scores compared to the positive control group (0.8 vs 2.4).

### Cytokine gene expression profile of the jejunum and ileum pre-challenge (day 23) and post-challenge (day 26)

**Figure 2:**
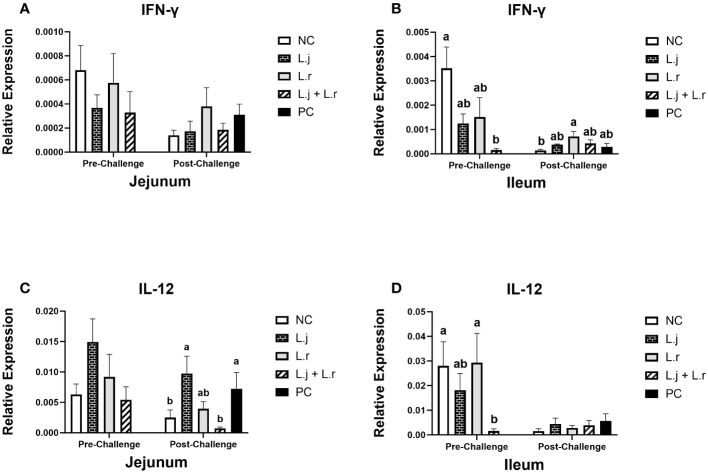
Gene Expression of IFN-γ and IL-12 Data represent the relative expression of IFN-γ **(A, B)** and IL-12 **(C, D)** in the jejunum and ileum before and after *C.perfringens* infection. Birds in groups 2 and 3 received *L. johnsonii* and *L. reuteri*, respectively, and group 4 received a probiotic cocktail containing both species. Groups 1 and 5 served as negative and positive control groups and did not receive lactobacilli. Birds in all groups (except the negative control) were orally challenged with *C.perfringens* on days 23 to 25 twice daily. On day 23 and 26 post-hatch, 6 birds per group were euthanized, and tissues were collected for gene expression analysis. Group means with no common alphabetical letters differ significantly. Error bars represent the standard error of the mean. Results were considered statistically significant if *P* < 0.05.

**Figure 3:**
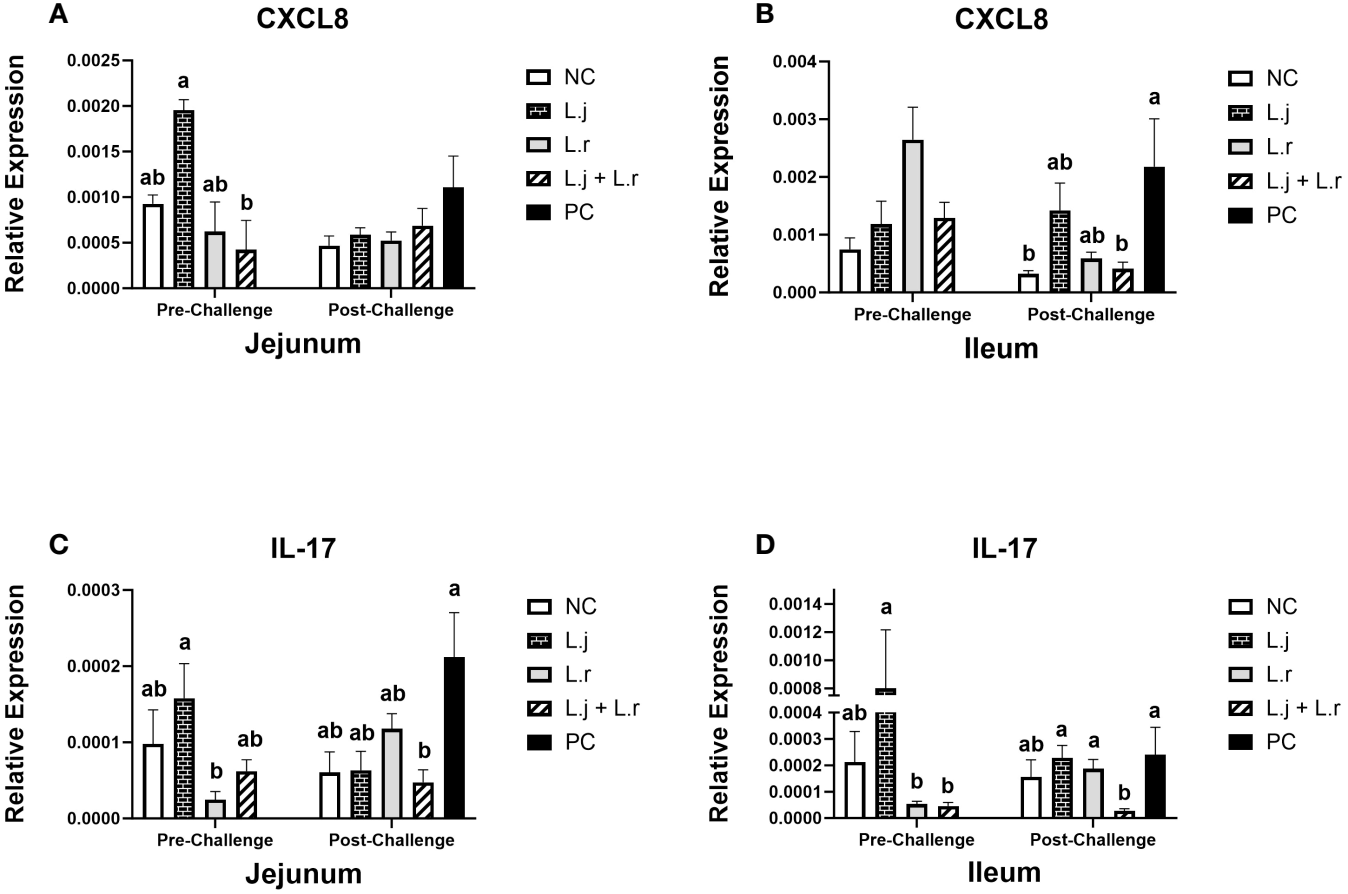
Gene Expression of CXCL8 and IL-17 Data represent the relative expression of CXCL8 **(A, B)** and IL-17 **(C, D)** in the jejunum and ileum before and after *C.perfringens* infection. Birds in groups 2 and 3 received *L. johnsonii* and *L. reuteri*, respectively, and group 4 received a probiotic cocktail containing both species. Groups 1 and 5 served as negative and positive control groups and did not receive lactobacilli. Birds in all groups (except the negative control) were orally challenged with *C.perfringens* on days 23 to 25 twice daily. On day 23 and 26 post-hatch, 6 birds per group were euthanized, and tissues were collected for gene expression analysis. Group means with no common alphabetical letters differ significantly. Error bars represent the standard error of the mean. Results were considered statistically significant if *P* < 0.05.

**Figure 4:**
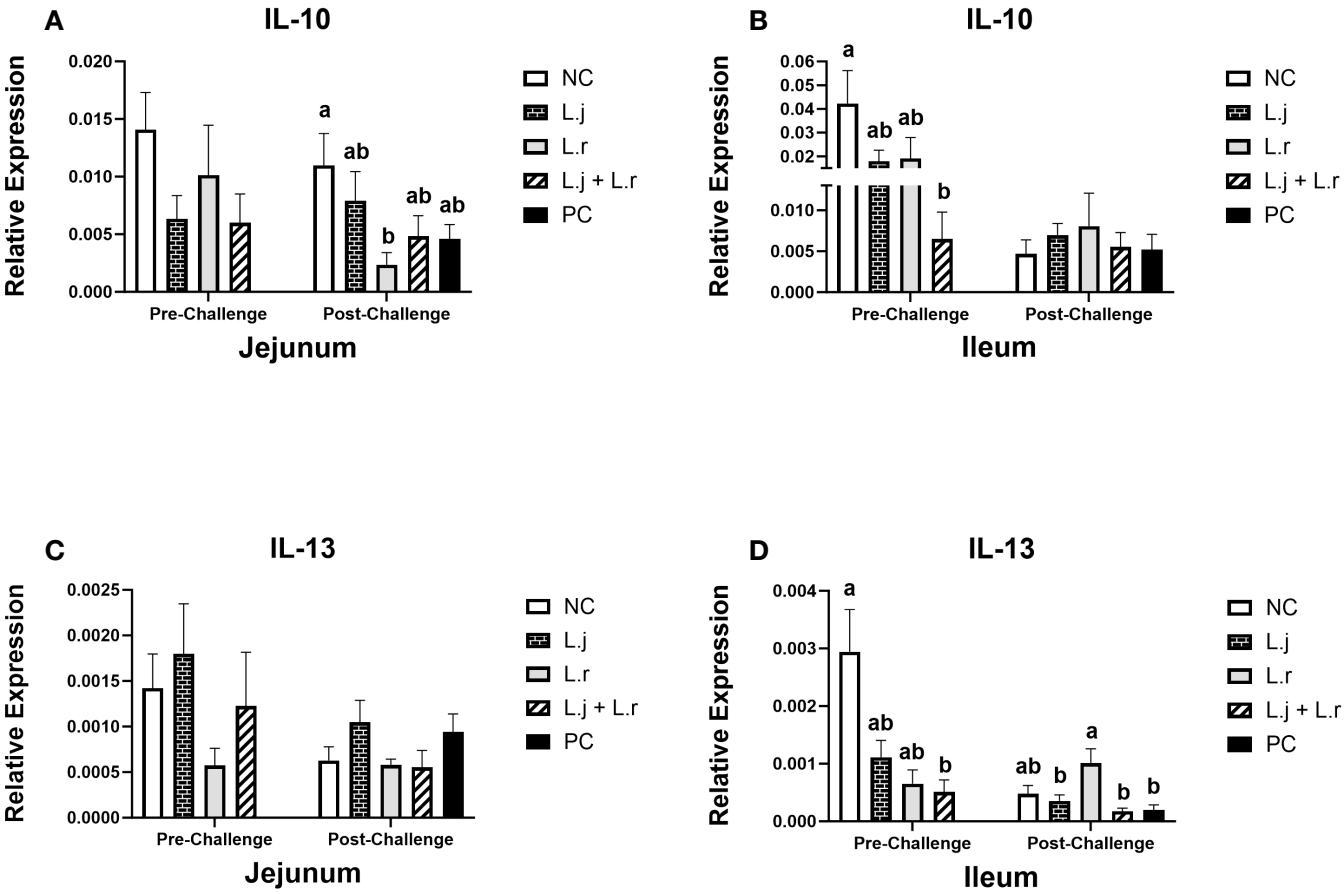
Gene Expression of IL-10 and IL-13 Data represent the relative expression of IL-10 **(A, B)** and IL-13 **(C, D)** in the jejunum and ileum before and after *C.perfringens* infection. Birds in groups 2 and 3 received *L. johnsonii* and *L. reuteri*, respectively, and group 4 received a probiotic cocktail containing both species. Groups 1 and 5 served as negative and positive control groups and did not receive lactobacilli. Birds in all groups (except the negative control) were orally challenged with *C.perfringens* on days 23 to 25 twice daily. On day 23 and 26 post-hatche, 6 birds per group were euthanized, and tissues were collected for gene expression analysis. Group means with no common alphabetical letters differ significantly. Group means with no common alphabetical letters differ significantly. Error bars represent the standard error of the mean. Results were considered statistically significant if *P* < 0.05.

The results for gene expression analysis in the intestine are presented in **Figures 2**–**4**. The expression of IFN-γ in the jejunum was not affected (*P* > 0.05) by lactobacilli treatment in pre- and post-challenge condition (**Figure 2A**). However, before CP infection, the expression of IFN-γ was reduced (*P* < 0.01) in the ileum of the group that was treated with the lactobacilli cocktail compared to the negative control group (untreated and unchallenged; **Figure 2B**). In addition, oral inoculation of *L. reuteri* enhanced (*P* < 0.05) the expression of IFN-γ compared to the negative control group post-infection.

Although the relative expression of IL-12 in the jejunum was not affected (*P* > 0.05) after lactobacilli treatment and prior to CP infection, the group that received the lactobacilli cocktail showed a decrease (*P* < 0.05) in the expression of IL-12 when compared to the negative control and the group that received *L. reuteri* alone (**Figures 2C, D**). Oral inoculation of the lactobacilli cocktail significantly reduced (*P* < 0.05) the expression of IL-12 in the ileum compared to the positive control group post-challenge, while no significant difference was observed in the ileum post-infection (**Figure 2D**).

On day 23 (pre-challenge), supplementation of chickens with *L. johnsonii* enhanced (*P* < 0.05) the expression of CXCL8 in the jejunum compared to the group that received the lactobacilli cocktail (**Figure 3A**). However, no significant difference (*P* > 0.05) was observed for CXCL8 expression in the ileum (**Figure 3B**). Post CP infection, the expression of CXCL8 was not affected (*P* > 0.05) in jejunum; however, the group that received the lactobacilli cocktail had significantly (*P* < 0.05) decreased expression of CXCL8 in the ileum compared to the positive control group (**Figures 3A, B**).

Oral inoculation of *L. johnsonii* significantly enhanced (*P* < 0.05) the expression of IL-17 compared to group that received *L. reuteri* in the jejunum and the ileum prior to CP challenge (**Figures 3C, D**). Furthermore, post CP-infection, the group that received the lactobacilli cocktail had significantly reduced (*P* < 0.05) expression of IL-17 (in both the jejunum and the ileum) compared to the positive control group (**Figures 3C, D**).

Prior to CP infection, the expression of IL-10 in the jejunum was not affected (*P* > 0.05) in different treatment groups (**Figure 4A**), while it was downregulated (*P* < 0.05) in the ileum in birds treated with the lactobacilli cocktail (**Figure 4B**). Oral treatment with *L. reuteri* downregulated (*P* < 0.05) the expression of IL-10 in the jejunum post-CP infection compared to the negative control group, while no significant difference (*P* > 0.05) was observed in the ileum (**Figures 4A, B**).

The expression of IL-13 was not affected (*P* > 0.05) in the jejunum before and after CP challenge (**Figure 4C**). However, in the ileum, before CP challenge, the expression of IL-13 was downregulated (*P* < 0.05) in the group that received the lactobacilli cocktail when compared to the negative control (**Figure 4D**). In addition, inoculation of birds with *L. reuteri* resulted in upregulation (*P* < 0.05) of the IL-13 expression in the ileum post CP-infection, compared to the other CP challenged groups (**Figure 4D**).

### Monocyte/macrophage and lymphocytes populations in jejunum and ileum

**Figure 5:**
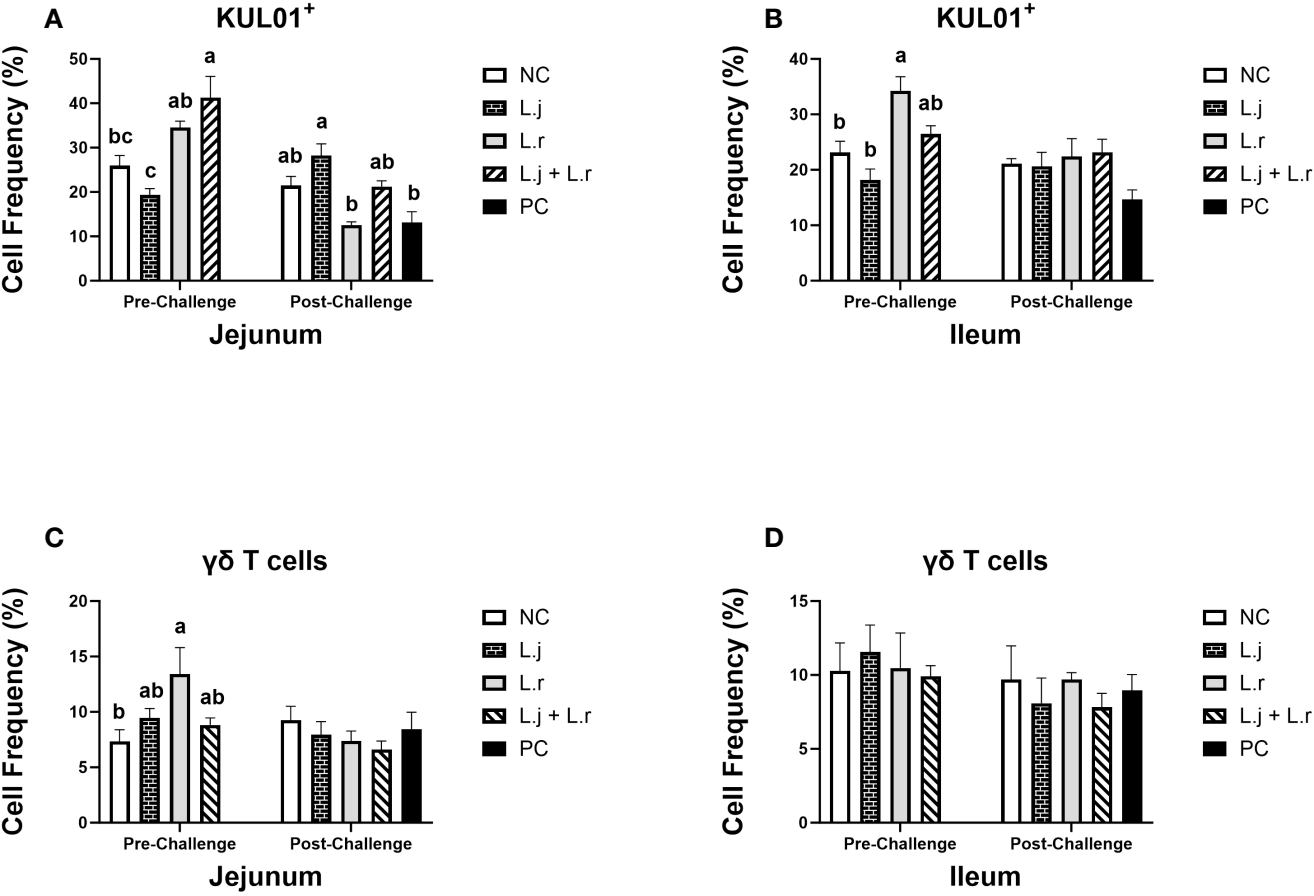
Frequency of Monocyte/macrophages and γδ T cells The frequency of monocyte/macrophages (KUL01^+^; **(A, B)** and γδ T cells **(C, D)** cells in the jejunum and ileum before and after *C.perfringens* infection. Birds in groups 2 and 3 received *L. johnsonii* and *L. reuteri*, respectively, and group 4 received a probiotic cocktail containing both species. Groups 1 and 5 served as negative and positive control groups and did not receive lactobacilli. Birds in all groups (except the negative control) were orally challenged with *C.perfringens* on days 23 to 25 twice daily. On day 23 and 26 post-hatch, 6 birds per group were euthanized, and tissues were collected for flow cytometry analysis. Group means with no common alphabetical letters differ significantly. Error bars represent the standard error of the mean. Results were considered statistically significant if *P* < 0.05.

**Figure 6:**
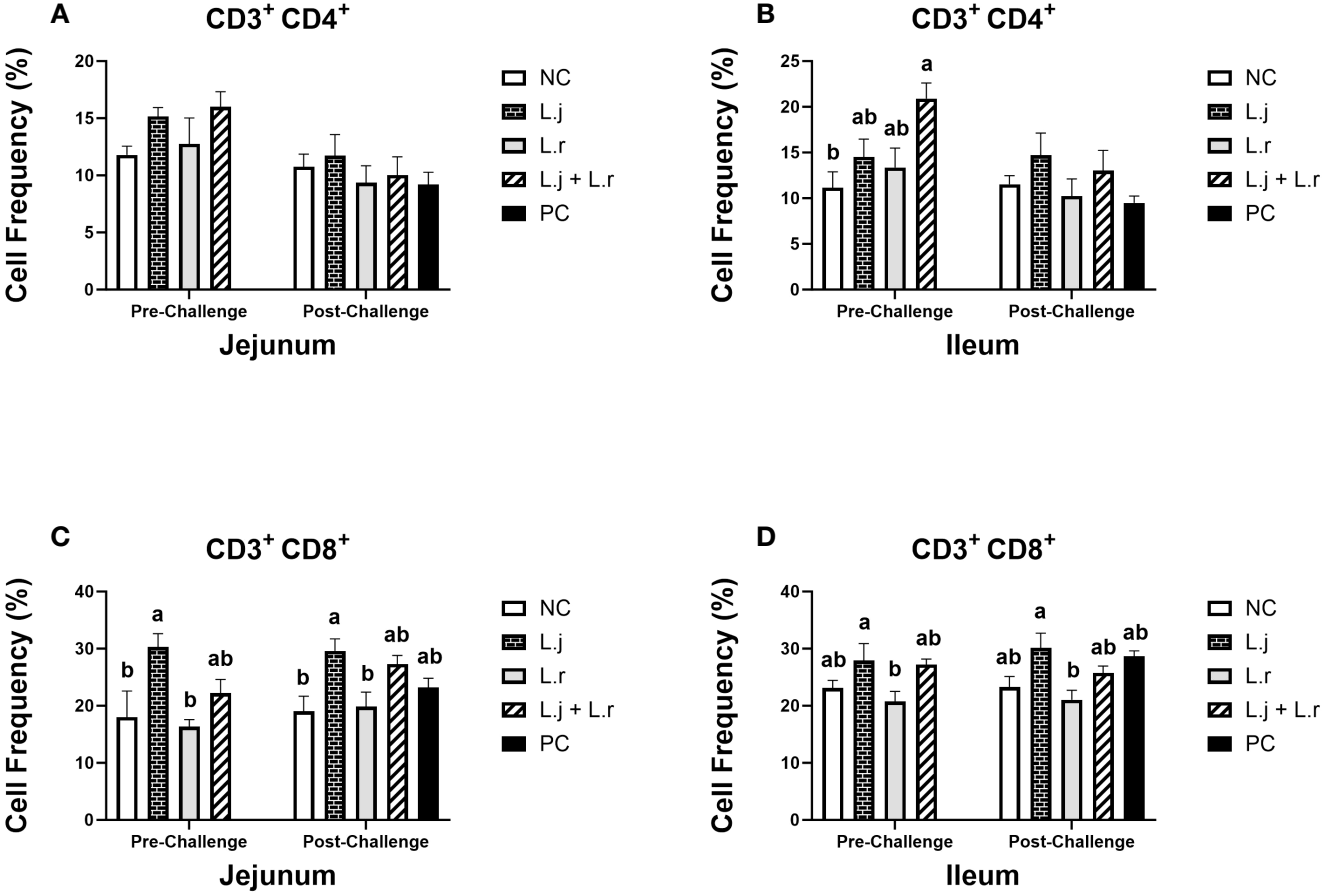
Frequency of CD3^+^CD4^+^ and CD3^+^CD8^+^ T cells The frequency of CD3^+^CD4^+^ **(A, B)** and CD3^+^CD8^+^ **(C, D)** T cells in the jejunum and ileum before and after *C.perfringens* infection. Birds in groups 2 and 3 received *L. johnsonii* and *L. reuteri*, respectively, and group 4 received a probiotic cocktail containing both species. Groups 1 and 5 served as negative and positive control groups and did not receive lactobacilli. Birds in all groups (except the negative control) were orally challenged with *C.perfringens* on days 23 to 25 twice daily. On day 23 and 26 post-hatche, 6 birds per group were euthanized, and tissues were collected for flow cytometry analysis. Group means with no common alphabetical letters differ significantly. Error bars represent the standard error of the mean. Results were considered statistically significant if *P* < 0.05.

**Figure 7:**
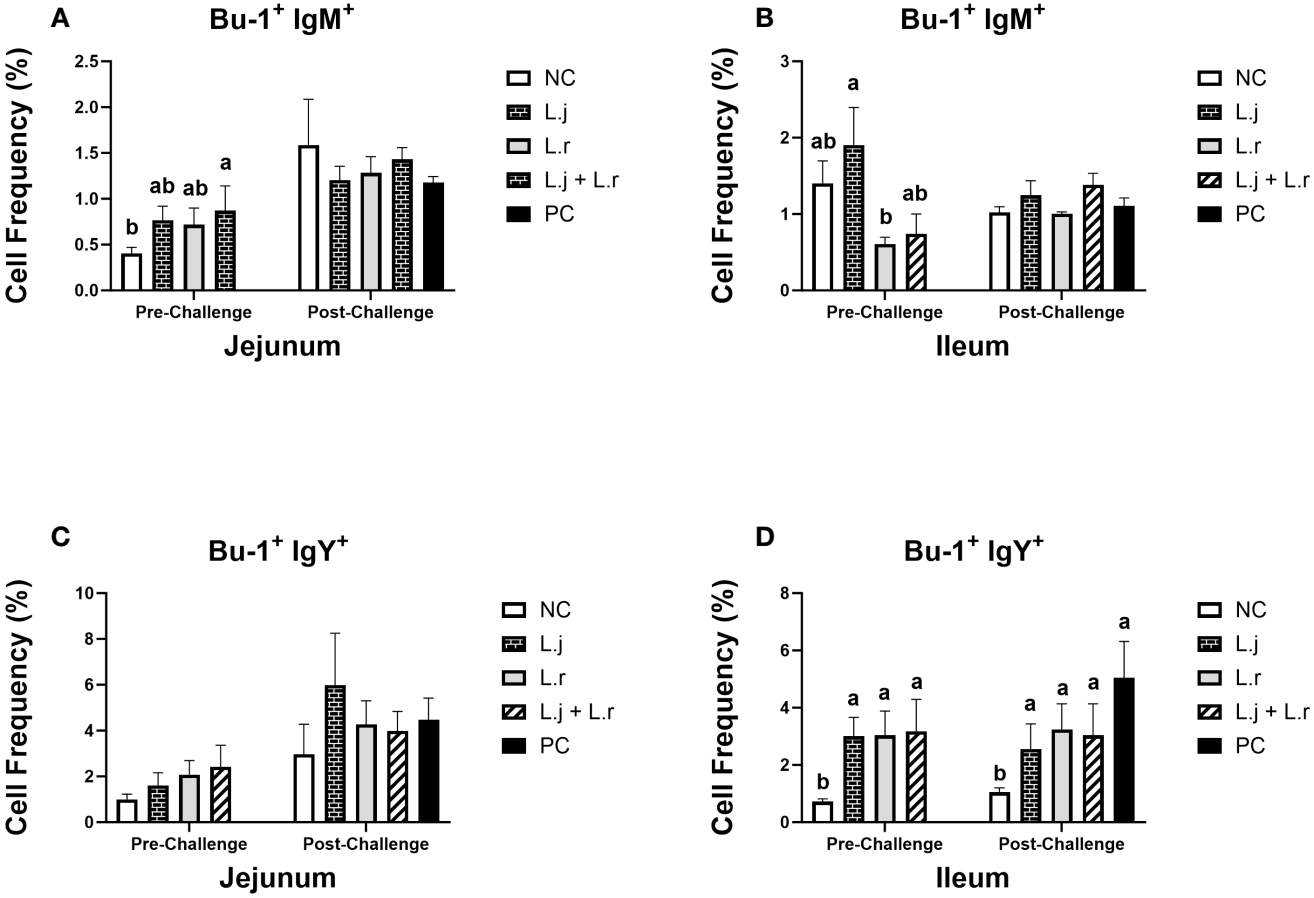
Frequency of Bu-1^+^ IgM^+^ and Bu-1^+^ IgY^+^ B cells The frequency Bu-1^+^ IgM^+^ **(A, B)** and Bu-1^+^ IgY^+^ **(C, D)** B cells in the jejunum and ileum before and after *C.perfringens* infection. Birds in groups 2 and 3 received *L. johnsonii* and *L. reuteri*, respectively, and group 4 received a probiotic cocktail containing both species. Groups 1 and 5 served as negative and positive control groups and did not receive lactobacilli. Birds in all groups (except the negative control) were orally challenged with *C.perfringens* on days 23 to 25 twice daily. On day 23 and 26 post-hatche, 6 birds per group were euthanized, and tissues were collected for flow cytometry analysis. Group means with no common alphabetical letters differ significantly. Error bars represent the standard error of the mean. Results were considered statistically significant if *P* < 0.05.

**Figure 8:**
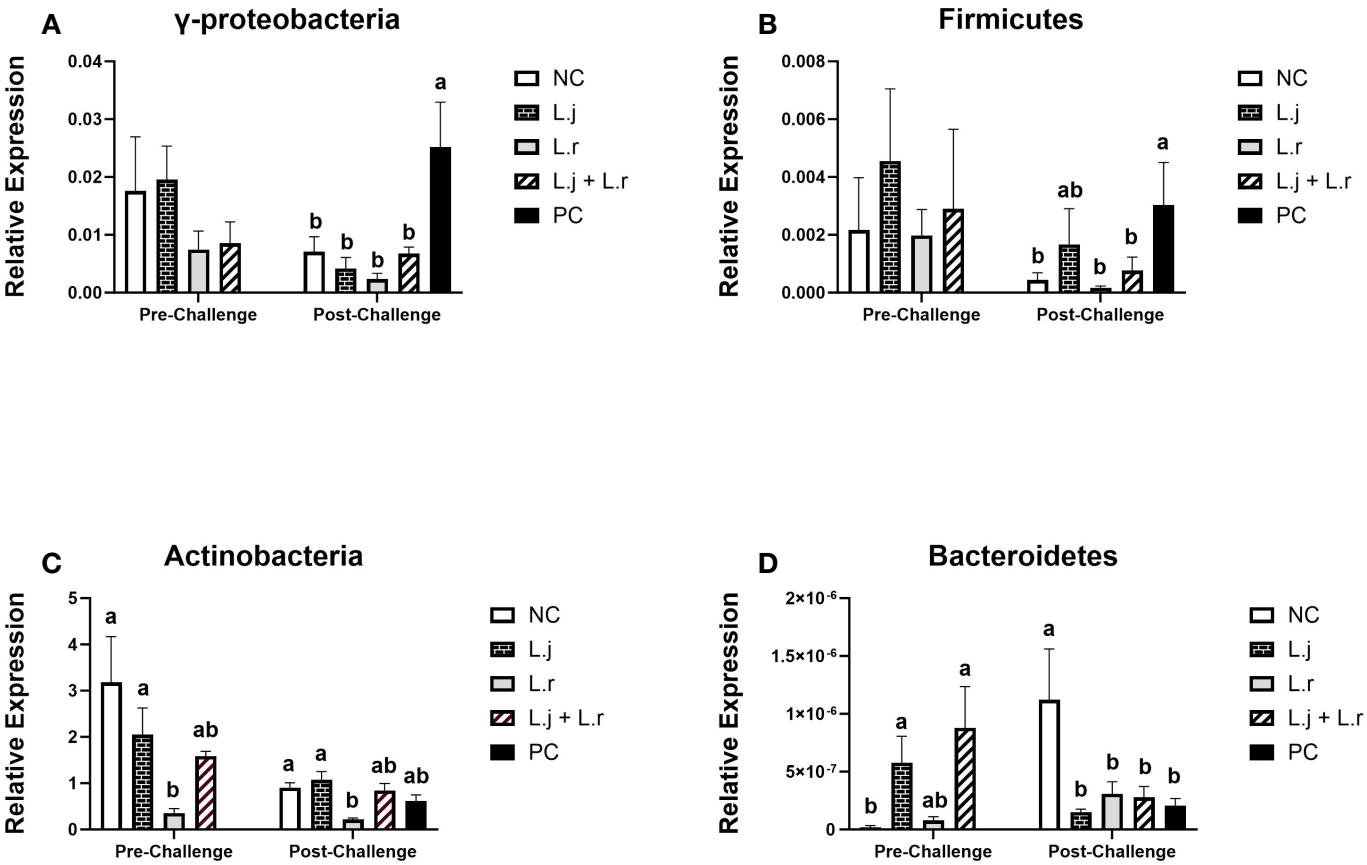
Relative Abundance of Cecal Microbiome at Phylum level Relative abundance of γ-Proteobacteria **(A)**, Firmicutes **(B)**, Actinobacteria **(C)**, and Bacteroidetes **(D)** before and after *C.perfringens* infection. Birds in groups 2 and 3 received *L. johnsonii* and *L. reuteri*, respectively, and group 4 received a probiotic cocktail containing both species. Groups 1 and 5 served as negative and positive control groups and did not receive lactobacilli. Birds in all groups (except the negative control) were orally challenged with *C.perfringens* on days 23 to 25 twice daily. On day 23 and 26 post-hatche, 6 birds per group were euthanized and cecal contents were collected for DNA extraction and PCR analysis. Group means with no common alphabetical letters differ significantly. Error bars represent the standard error of the mean. Results were considered statistically significant if *P* < 0.05.

The results for intestinal immune system cell populations are shown in **Figures 5**–**8**. Prior to challenge, the group that received the lactobacilli cocktail had a significantly enhanced (*P* < 0.05) percentage of monocytes/macrophages compared to the non-treated and *L. johnsonii*-treated groups in the jejunum (**Figure 5A**). In the ileum, birds that were treated with *L. reuteri* had higher frequency (*P* < 0.05) of monocytes/macrophages compared to the non-treated birds and the ones that were treated with *L. johnsonii* (**Figure 5B**). Post-infection, treatment of chickens with *L. reuteri* increased (*P* < 0.05) the frequency of monocytes/macrophages compared to the positive control group and group that received *L. johnsonii* in the jejunum (**Figure 5A**). However, the frequency of monocytes/macrophages was not affected (*P* > 0.05) by treatment or CP infection in the ileum (**Figure 5B**).

The frequency of γδ T cells was elevated (*P* < 0.05) in group that received *L. reuteri* compared to the negative control group in jejunum before CP infection, while no significant difference (*P* > 0.05) was observed after CP challenge (**Figure 5C**). The percentage of γδ T cells was not affected (*P* > 0.05) by lactobacilli treatment pre- and post-challenge in ileum (**Figure 5D**).

The percentage of CD3^+^CD4^+^ T cells in the jejunum was not affected (*P* > 0.05) with treatment groups pre- and post-challenge (**Figure 6A**). In the ileum, on the other hand, treatment of birds with the lactobacilli cocktail increased (*P* < 0.05) the frequency of CD3^+^CD4^+^ T cells compared to the non-treated group before challenge (**Figure 6B**). Post CP-infection, no significant difference (*P* > 0.05) was observed in both the jejunum and ileum (**Figure 6B**). The frequency of CD3^+^CD8^+^ T cells was enhanced (*P* < 0.05) in the group that received *L. johnsonii* when compared to the negative control and *L. reuteri*-treated groups in the jejunum prior to and post CP challenge (**Figure 6C**). In addition, inoculation of birds with *L. johnsonii* increased (*P* < 0.05) the percentage of CD3^+^CD8^+^ T cells compared to the *L. reuteri*-treated group in ileum before and after CP challenge (**Figure 6D**).

Oral inoculation of the lactobacilli cocktail enhanced (*P* < 0.05) the frequency of Bu-1^+^IgM^+^ B cells in the jejunum prior to CP challenge (**Figure 7A**). However, the number of these cells was not altered (*P* > 0.05) by lactobacilli treatment post-infection. In the ileum, treatment of chickens with *L. johnsonii* significantly enhanced (*P* < 0.05) the number of Bu-1^+^IgM^+^ B cells compared to the *L. reuteri*-treated group before CP challenge. However, no significant difference was observed (*P* > 0.05) after CP infection (**Figure 7B**).

The percentage of Bu-1^+^IgY^+^ B cells was not affected (*P* > 0.05) by treatment groups in the jejunum before or after CP challenge (**Figure 7C**). All lactobacilli-treated groups had significantly enhanced (*P* < 0.05) frequencies of Bu-1^+^IgY^+^ B cells in the ileum prior to and post CP challenge when compared to the negative control group (**Figure 7D**). The percentage of Bu-1^+^IgA^+^ B cells was not altered (*P* > 0.05) by treatment group in the jejunum before and after CP challenge (**Figure 7D**). However, inoculation of birds with *L. johnsonii* increased (*P* < 0.05) the number of Bu-1^+^IgA^+^ B cells in the ileum when compared to the negative control group post CP infection (**Figure 7D**).

### Cecal microbial communities

The results for cecal microbiome communities are presented in **Figure 8**. Relative abundance of the phyla Firmicutes and γ-Proteobacteria were not affected (*P* > 0.05) with lactobacilli treatment prior to CP infection (**Figures 8A, B**). However, after CP challenge, all lactobacilli-treated groups showed reduced relative abundance of γ-Proteobacteria (*P* < 0.01), and a significant decrease (*P* < 0.05) in abundance of Firmicutes was observed in groups that were treated with *L. reuteri* and the lactobacilli cocktail when compared to the positive control (**Figures 8A, B**). Oral inoculation of birds with *L. reuteri* significantly reduced (*P* < 0.05) the relative abundance of Actinobacteria compared to the *L. johnsonii*-treated and negative control groups before and after CP challenge (**Figure 8C**). Treatment of chickens with *L. johnsonii* and the lactobacilli cocktail enhanced (*P* < 0.01) the abundance of the phylum Bacteroidetes compared to the negative control group prior to CP infection. Relative abundance of the phyla Bacteroidetes was significantly reduced (*P* < 0.05) in all challenged groups compared to the negative control group post-challenge (**Figure 8D**).

## Discussion

Necrotic enteritis is an important intestinal disease in broiler chickens that is characterized by overgrowth of CP and production of various toxins that impair the intestinal mucosa, causing inflammation and hemorrhages (7). Interaction of toxins secreted by CP (such as α-toxin, NetB, and TpeL) and intestinal epithelial cells, intraepithelial lymphocytes, and immune system cells in the lamina propria can lead to potent pro-inflammatory responses (6). Therefore, regulation of immune system cell responses that are related to inflammation in the intestinal mucosa is likely essential for protection against NE.

Overall, the results for gross pathology demonstrated that treatment with the lactobacilli cocktail showed the strongest antagonistic activity against CP as demonstrated by significantly lower mean lesion scores in the small intestine. This result suggests a synergistic or additive interaction between the two lactobacillus species against CP-induced NE. In the support of this observation, lactobacilli treatment reduced the expression of inflammatory of cytokines in the intestine and restored the composition of gut microbiome of chicken after CP infection.

Given the critical role of cytokines and chemokines in communication between gut epithelial cells and immune system cells and in regulation of innate and adaptive responses (24), it is likely that cytokines and chemokines play a key role in the chicken response to necrotic enteritis caused by CP. In the present study, prior to challenge, treatment of chickens with a cocktail of lactobacilli significantly reduced the expression of IFN-γ in the ileum compared to the negative control group. Considering the role of IFN-γ in activation of antigen presenting cells (25), downregulation of this cytokine in the group treated with lactobacilli suggests immunoregulatory activities of these bacterial species. In addition to IFN-γ, the expression of IL-12 was downregulated in the ileum of the group treated with the lactobacilli cocktail when compared to the negative control group. IL-12 is a T-helper I cytokine that has been shown to induce intestinal inflammation in an IFN-γ–dependant manner in mice (26). Therefore, downregulation of IL-12 in the lactobacilli cocktail-treated group is in line with the observed decrease in expression of IFN-γ. This IL-12–mediated decrease in IFN-γ also could have led to decreased inflammation in the chicken intestine in the present study, supporting the observation of reduced intestinal lesion scores in chickens treated with the lactobacilli cocktail. Nevertheless, a previous study by our group showed that a different lactobacilli cocktail upregulated the expression of IFN-γ and IL-12 in the jejunum of chickens (20). This could be related to the species-specific activity of lactobacilli suggesting that various lactobacillus species may exert different cytokine expression patterns. No significant difference was observed for IFN-γ expression in the jejunum and ileum after CP infection for all challenged groups. Similarly, Fasina and Lillehoj (27) demonstrated that the expression of IFN-γ in the intestine was not affected by CP infection.

Prior to CP challenge, the expression of both IL-10 and IL-13 in the ileum was downregulated in the lactobacilli cocktail-treated group when compared to the negative control group. IL-10 and IL-13 both have anti-inflammatory roles and suppress inflammatory responses by reducing the production of pro-inflammatory cytokines such as IFN-γ and IL-12 (28, 29). Notably, both groups treated with a single lactobacilli species demonstrated a reduction in IL-10 and IL-13 expression prior to CP challenge; however, reduction was most pronounced in the group treated with the lactobacilli cocktail, suggesting an additive or synergistic effect on the expression of these cytokines. Considering that the expression of inflammatory cytokines was also downregulated in the lactobacilli cocktail-treated group in the present study, lower expression of anti-inflammatory cytokines in this group might be explained by balance between the pro- and anti-inflammatory cytokines.

In addition, changes in some cytokine expression in the present study support a reduced inflammatory response in chickens treated with the lactobacilli cocktail. For example, post-CP infection, chickens that were treated with the lactobacilli cocktail demonstrated a significant reduction in the expression of CXCL8 in the ileum compared to the positive control group. CXCL8 is a pro-inflammatory cytokine produced by a variety of cells including macrophages and epithelial cells and plays a key role in inflammatory responses by recruitment and activation of neutrophils to the site of inflammation (30). In addition to CXCL8, the expression of IL-17 in the lactobacilli cocktail-treated group was significantly downregulated in both the jejunum and ileum following CP infection when compared to the positive control group. IL-17 is a potent inflammatory cytokine produced by T-helper 17 cells and plays an important role in host defense by attracting macrophages and neutrophils to the infection site and by stimulating the production of different proinflammatory cytokines (31). It has also been reported that CP infection enhances the expression of IL-17 in the intestine of broiler chickens (27). Therefore, reduced expression of CXCL8 and IL-17 in the group that received the lactobacilli cocktail further supports the anti-inflammatory roles of these probiotic bacteria in response to CP infection.

Evidence suggests that NE can modify frequencies of immune system cells in the intestine of chickens (18, 20). In the current study, a change in the number of immune system cells following CP infection was observed. Considering the role of probiotics in the regulation of intestinal epithelial cells and immune system cells, effects of treatment with L. *johnsonii* and L. *reuteri* on the frequency of monocyte/macrophages and lymphocytes in the small intestine were evaluated. The results of flow cytometry analysis revealed that prior to CP challenge, the group that received the lactobacilli cocktail had significantly enhanced numbers of monocytes/macrophages (KU0L1^+^) in the jejunum. Furthermore, treatment of chickens with *L. reuteri* significantly increased the number of KU0L1^+^ cells in the ileum compared to the negative control. Similar to the present study, Higgins and colleagues (32) found that lactobacilli treatment enhances the percentage of KU0L1^+^ cells in the intestine of broiler chickens. The elevated number of intestinal macrophages observed in the lactobacilli-treated groups is highly relevant for CP infection, given the important role of macrophages in host defence (33, 34). Notably, intestinal macrophages display immunoregulatory activities that can suppress inflammation and maintain intestinal homeostasis (35). It remains to be determined whether the lactobacilli-induced macrophages in the present study played a role in regulating inflammatory responses during CP infection; however, the observed reductions in inflammatory cytokines and intestinal lesions in the lactobacilli cocktail-treated group support this suggestion.

The analysis of T cell populations in the intestine prior to CP challenge demonstrated that lactobacilli treatment likely played a role in recruitment of T cells to the intestine. For example, the frequency of CD3^+^CD4^+^ T cells was elevated in the lactobacilli cocktail-treated group when compared to the negative control group in the ileum. In addition, treatment with *L. johnsonii* enhanced the frequency of CD3^+^CD8^+^ T cells in both the jejunum and ileum prior to CP challenge compared to the negative control group. Similarly, Wang and colleagues (18) demonstrated that treatment of chickens with *L. johnsonii* increased the abundance of CD3^+^CD4^+^ and CD3^+^CD8^+^ T cells in the ileum. It has also been shown that oral inoculation of a lactobacilli cocktail containing *L. acidophilus* and *L. reuteri* enhanced the number of CD4^+^ and CD8^+^ cells in the small intestine of chickens (36). The higher frequency of T cells in lactobacilli-treated groups suggests that lactobacilli treatment may have played a role in the recruitment of T cells subsets to the intestine or propagation of resident T cells in the intestine; however, their role in the present study in host defense against CP infection remains to be elucidated.

Populations of B cells in the present study showed that all three lactobacilli-treated groups had enhanced numbers of IgY^+^ B cells in the ileum before and after challenge with CP. In addition, chickens that received the lactobacilli cocktail had a higher frequency of Bu-1^+^IgM^+^ cells prior to CP challenge in the jejunum when compared to the negative control group. A pervious study in our group also demonstrated that lactobacilli treatment increased B cell frequency in the cecal tonsils of chickens (20). Nevertheless, other studies have shown that lactobacilli administration had no effect on Bu-1 mRNA expression levels in the chicken intestine (37, 38). In addition to antibody producing activity of B cells, they can act as professional antigen presenting cells by phagocytosis, processing and presenting antigens to T helper cells. Therefore, the higher frequency of B cells in the intestine of the lactobacilli treated birds may point to some of the immunomodulatory activities of L. *johnsonii* and L. *reuteri*, leading to the recruitment of B cells to the chicken intestine. CP infection is understood to induce gut dysbiosis in chickens, and the level of dysbiosis has been directly linked to the severity of necrotic enteritis and inflammation in the intestine (39, 40). Probiotics have been shown to maintain gut homeostasis and restore disrupted gut microbiota caused by CP infection (41). The results of microbiome analysis in the present study showed that prior to CP challenge, inoculation of birds with *L. johnsonii* and the lactobacilli cocktail enhanced the relative abundance of the phyla Bacteroidetes. Intestinal Bacteroidetes are considered one of the main butyrate producing phyla (42). Butyrate is a short chain fatty acid that has been shown to improve gut health by decreasing intestinal inflammation and oxidative stress (43). Therefore, a higher relative abundance of Bacteroidetes in lactobacilli-treated groups suggests a beneficial effect of lactobacilli in reducing intestinal inflammation.

In addition, here, we demonstrated that challenging the birds with CP was associated with a shift in the cecal microbiota and significantly increased the relative abundance of the phyla Firmicutes and γ-Proteobacteria in the positive control group compared to the lactobacilli-treated group and negative control group. These results demonstrated that treatment of birds with lactobacilli decreases the relative abundance of the phyla Firmicutes and γ-Proteobacteria compared to the positive control group after CP infection suggesting the role of lactobacilli in maintaining of gut microbiota composition after CP infection. Lin and colleagues (44) also demonstrated that treatment with probiotics alleviated disruption in the cecal microbiota of chickens challenged with CP. However, further detailed analysis comprising metagenomic and metatranscriptomic approaches are required to assess the impact of lactobacilli on CP modulation of the chicken gut microbiota.

In conclusion, treatment of birds with a probiotic cocktail containing both *L. johnsonii* and *L. reuteri* led to significantly reduced mean lesion scores in the intestine following CP challenge. The impact of lactobacilli administration was also evident when considering intestinal gene expression, which revealed downregulation of multiple proinflammatory genes including CXCL8, IL-12, and IL-17. In addition, chickens treated with lactobacilli demonstrated increased quantities of lymphocytes and macrophages in the intestine. In addition to these immunomodulatory findings, treatment with lactobacilli maintained the cecal microbiota of chickens exposed to CP challenge. however, further analysis is required to explore the impacts of lactobacilli on gut microbiota composition following CP infection.

## Data availability statement

The original contributions presented in the study are included in the article/**Supplementary Material**. Further inquiries can be directed to the corresponding author.

## Ethics statement

The animal study was approved by University of Guelph Animal Care Committee. The study was conducted in accordance with the local legislation and institutional requirements.

## Author contributions

MA: Conceptualization, Data curation, Formal Analysis, Investigation, Methodology, Project administration, Software, Supervision, Validation, Visualization, Writing – original draft, Writing – review & editing. BS: Conceptualization, Data curation, Formal Analysis, Investigation, Methodology, Project administration, Software, Supervision, Validation, Visualization, Writing – review & editing. NB: Formal Analysis, Investigation, Methodology, Software, Writing – review & editing. SR: Investigation, Methodology, Writing – review & editing. SS: Conceptualization, Funding acquisition, Project administration, Resources, Supervision, Validation, Visualization, Writing – review & editing.
