# Supplementary material for: Molecular and cellular characterization of immunity conferred by lactobacilli against necrotic enteritis in chickens
Source: Front Immunol. 2023 Nov 7;14:1301980. doi: 10.3389/fimmu.2023.1301980 (PMC10662302; doi:10.3389/fimmu.2023.1301980)
Supplement: Supplementary file 1 [file DataSheet_1.pdf]

**Supplementary Material 1: Gating Strategies**

Cells were gated using forward scatter (FSC)-A and side scatter (SSC)-A to eliminate debris and clumped cells based on the size and granularity of the cells. Single cells were sub-gated using FSC-A and FSC-H. Dead cells were excluded using a live/dead marker (7-AAD). For panel A, mouse anti-chicken monocyte/macrophage-FITC (KUL01) was used as a marker for monocyte/macrophage-FITC (KUL01). Mouse anti-chicken TCRγδ-PE (TCR-1) and mouse anti-chicken CD3-PB were used to determine $CD3^+$ γδ T cells and $CD3^+$ αβ T cells respectively. Then, $CD3^+$ αβ T cells were sub-gated using mouse anti-chicken CD4-PE-Cy7 and mouse anti-chicken CD8-APC to differentiate $CD4^+$ and $CD8^+$ T cells respectively.

For panel B, cells were gated using forward scatter (FSC)-A and side scatter (SSC)-A. Single cells were sub-gated using FSC-A and FSC-H. Dead cells were excluded using a live/dead marker (7-AAD). Mouse anti-chicken Bu-1-PB was used as a marker for B cells. Then, Bu-$1^+$ cells were sub-gated using mouse ani-chicken IgY-FITC, and mouse anti-chicken IgM-APC to determine IgY+ and IgM+ B cells, respectively.
