# Supplementary figures and images for: Molecular and cellular characterization of immunity conferred by lactobacilli against necrotic enteritis in chickens
Source: Front Immunol. 2023 Nov 7;14:1301980. doi: 10.3389/fimmu.2023.1301980 (PMC10662302; doi:10.3389/fimmu.2023.1301980)

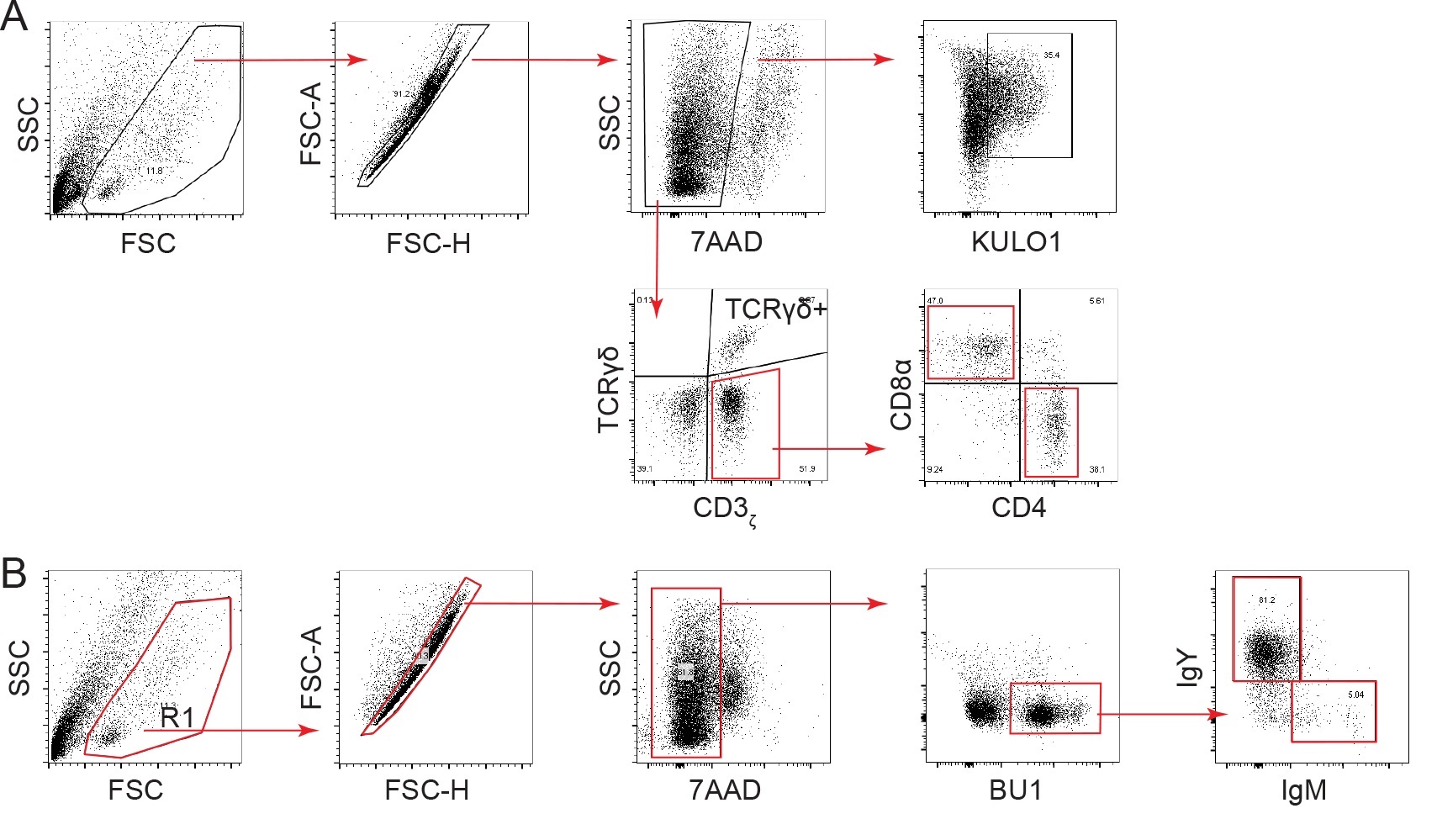

Supplement: Supplementary file 2 [file Image_1.jpeg]
